# Serious games for serious crises: reflections from an infectious disease outbreak matrix game

**DOI:** 10.1186/s12992-020-00547-6

**Published:** 2020-03-10

**Authors:** Julia Smith, Nathan Sears, Ben Taylor, Madeline Johnson

**Affiliations:** 1 Faculty of Health Sciences, Simon Fraser University, Blusson Hall, Room 11802, 8888 University Drive, Burnaby, BC V5A 1S6 Canada; 2 Political Science, University of Toronto, Toronto, Canada; 3 Department of National Defense, Government of Canada, Ottawa, Canada; 4 Global Affairs Canada, Government of Canada, Ottawa, Canada

**Keywords:** Games, Training, Outbreaks, Health security, Gender

## Abstract

**Background:**

While there is widespread recognition of global health failures when it comes to infectious disease outbreaks, there is little discussion on how policy-makers and global health organizations can learn to better prepare and respond. Serious games provide an underutilized tool to promote learning and innovation around global health crises. In order to explore the potential of Serious Games as a policy learning tool, Global Affairs Canada, in collaboration with the Department of National Defense and academic partners, developed and implemented a matrix game aimed at prompting critical reflection and gender-based analysis on infectious disease outbreak preparedness and response. This commentary, written by the core development team, reflects on the process and outcomes of the gaming exercise, which we believe will be of interest to others hoping to promote innovative thinking and learning around global health policy and crisis response, as well as the application of serious games more broadly.

**Main body:**

Participants reported, through discussions and a post-game survey, that they felt the game was reflective of real-world decision-making and priority-setting challenges during a crisis. They reflected on the challenges that emerge around global health co-operation and outbreak preparedness, particularly noting the importance of learning to work with private actors. While participants only sporadically applied gender-based analysis or considered the social determinants of health during the game, post-game discussions led to reflection on the ways in which equity concerns are put aside during a crisis scenario and on why this happens, offering critical learning opportunities.

**Conclusion:**

Matrix games provide opportunities for policy-makers and health professionals to experience the challenges of global health co-operation, test ideas and explore how biases, such as those around gender, influence policy-making and implementation. Due to their flexibility, adaptability and accessibility, serious games offer a potentially powerful learning tool for global health policy-makers and practitioners.

## Background

The first annual report of the Global Preparedness Monitoring Board, convened by the World Health Organization and World Bank, notes that the world is ill prepared to respond to future outbreaks [1]. The validity of this statement has unfortunatly been confirmed by both the Ebola outbreak in the Democratic Republic of the Congo and COVID-19 outbreak in China. Critics of current and past outbreak responses highlight the need to consider political and security, as well as biological, threats to effective responses [2], and the consequences of failing to address socio-economic and cultural dynamics, such as those related to gender inequities, within health crises [3]. While there is widespread recognition of policy failures during outbreaks, there is little discussion on how policy-makers, global health organizations and health professionals can better prepare and respond [4].

Clearly, it is difficult to learn ahead of time how to address the unpredictable and ever-shifting milieu of biological, socio-economic and political factors that determine an outbreak. Actors have little time to consider the multiple consequences of decisions and are hesitant to implement risky innovations during high-profile crises [5]. In particular, structural drivers of outbreaks, such as gender inequities and other social determinants of health, are often neglected in favour of addressing immediate biological threats with technical fixes. But what if policy-makers and practitioners could gain prior insight into the intended and unintended consequences of decision-making? What if they could test out innovative ideas in a safe environment? What if they could practice applying gender-based analysis?

Interested in the potential of ‘serious games’ as a tool for learning and planning, Global Affairs Canada (GAC), in collaboration with the Department of National Defense (DND) and academic partners, developed and implemented a ‘matrix game’ in March 2019 on an international response to a global health crisis from an infectious disease outbreak. The design and implementation of the game was based on the following objectives:

- to assess the utility of (matrix) games as a foreign policy learning tool, especially for crisis scenarios;
- to encourage critical thinking about preparedness for a global pandemic scenario; and
- to promote the application of gender-based analysis (“GBA+”) in decision-making.

This commentary, written by the core development team, reflects on the process and outcomes of this gaming exercise. Rather than an experiment or research project, the game was designed to demonstrate the ways in which such tools might inform global health policy and practice. We hope our findings will be of interest to those looking to promote innovative thinking and learning around global health policy and crisis response.

## Methods

Game participants were recruited voluntarily via an email invitation sent out to GAC and DND employees, who were encouraged to pass on the invitation to others they thought might be interested. Eighteen individuals participated, including eight from GAC, seven from DND, one graduate student and two non-governmental organization representatives. Ten participants were men and eight were women. Participants had a variety of levels of experience with, and knowledge of, global health crises, with some having played leadership roles in related programs and others having no previous direct experience.

For this commentary, analyzing the gaming exercise, we draw on two sources of information. First, we conducted participant observations of the game play and a debriefing discussion. Participants (18) were informed prior to the game that we would be anonymously recording statements, plays and observations. An informed consent form providing permission for participant observation as a source of data collection was distributed, which all participants signed and returned. Second, we collected data through a post-game survey, circulated via email to all participants 3 days after the game. The survey included both Likert scale and open answer questions about participants’ experience of and learnings from the game. Fourteen out of 18 (78%) surveys were returned.

Results were analyzed through a reflexive iterative process, where each author reviewed their notes and reflected on quantitative and qualitative survey responses. Key findings and outcomes were then identified through reflexive team discussions. These were organized in relation to the primary objectives of the gaming exercise, with illustrative survey results related to each finding selected by the lead author and reviewed by the co-authors.

## Main text

### Serious games

While “games” are popularly recognized for their entertainment value (e.g., board-games and video-games), they can be more generally understood as simulated environments in which players make decisions and interact in ways that shape outcomes. “Serious games” refer to the use of games as a method to promote better human understanding and decision-making with respect to some phenomenon. Militaries around the world have used war-games of various kinds since the nineteenth century, largely for training, but also for analytical purposes [6]. More recently, the applications of serious games have expanded to other domains, especially in contexts where human decision-making and interactions are shaped by complex social, technological and/or environmental systems [7].

Most serious games are based on simplified models of a real—or potentially real—situation (a “scenario”), which enable participants to gain firsthand experience in making decisions and interacting with other actors in a dynamic environment [8]. While games are not usually meant to be predictive, they offer opportunities for participants to think about issues in contexts that can be conducive to, inter alia, identifying and thinking critically about assumptions, generating new ideas and insights, knowledge exchange and discussion, and ‘experiential learning’ [8]. There are a variety of game types, ranging from games with rigid rules and procedures and/or high-degrees of complexity in technical details (often hosted on computer systems), through to those with more flexible structures and lower complexity (e.g. unstructured role-plays).

“Matrix games” are a form of semi-structured games, in which there is a scenario that develops over the course of a number of rounds, and multiple players who implement decisions by proposing an action with a specific objective (an “argument”), and various reasons why this action should be successful. The argument is then assessed by other participants, who may propose additional reasons why the action should be (un) successful, and then adjudicated by a facilitator (or “gamemaster”), who assigns a probability of success and rolls dice to determine the outcome. For example, if an action is deemed likely to succeed, a roll of four or higher on two dice might be required; if it was unlikely to succeed, a nine or higher might be required. The outcomes of (un) successful actions influence the development of the scenario. The section below describes a matrix game that was developed and implemented at GAC in March 2019 in order to explore preparedness and gender-based analysis in an international response to a global health crisis.

### Game design: Pangea 2030

**Fig. 1:**
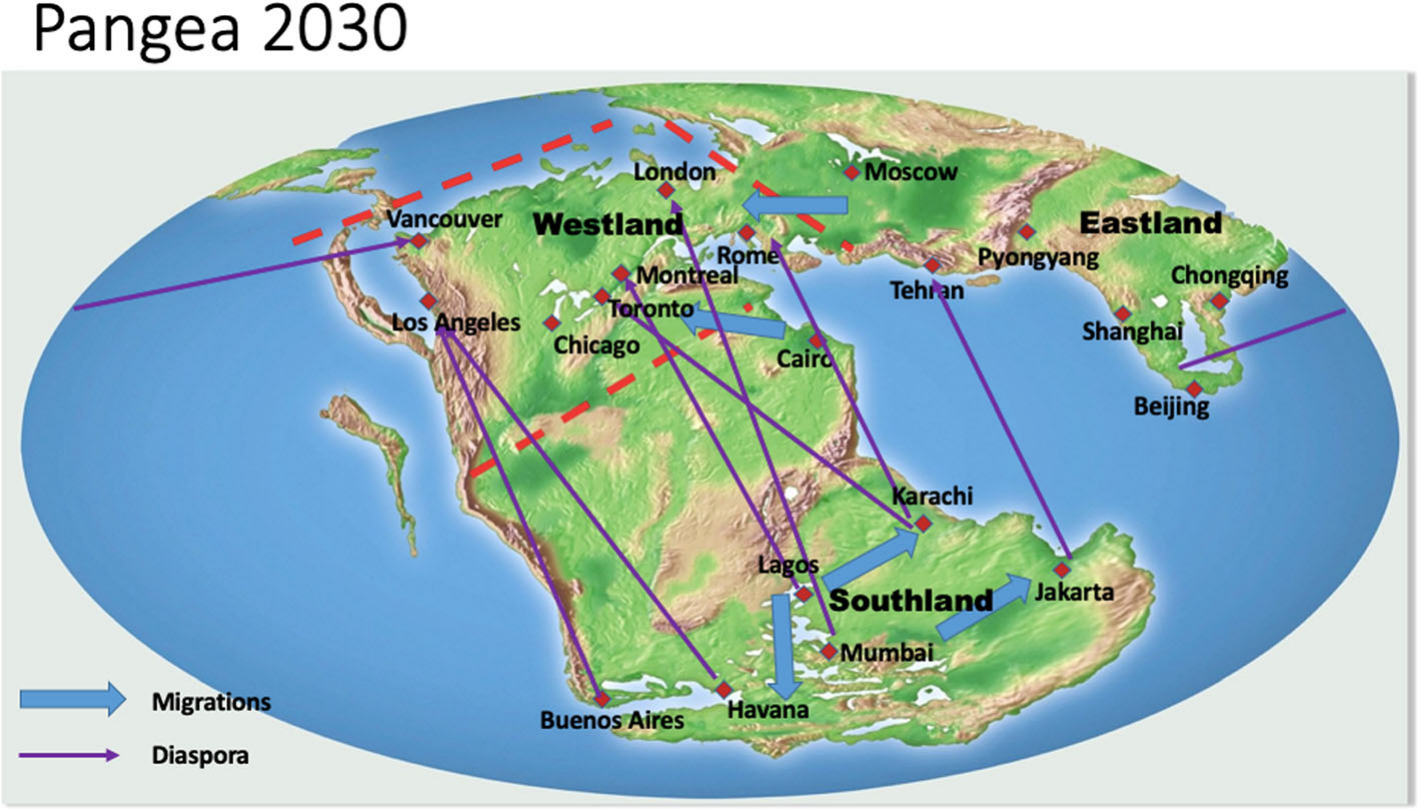
Pangea 2030

The game was set in an alternative world, “Pangea”,^1^ in the year 2030 (see Fig. 1). While many of the issues, tensions and some place names are reflected the real-world, creating an alternative setting allowed us to abstract away from some of the complexity of real-world events and actors. Pangea included three regions: Westland, Eastland and Southland. Supporting maps indicated the alignment of the regions, the location of major conurbations and border characteristics, as well as trade, travel and migration routes.

Game participants were divided into six familiar, but fictional actors: a state-like actor for each of the three regions (‘Westland’, ‘Eastland’ and ‘Southland’); the ‘Global Health Organization’, a multilateral organization with the mission to lead global health responses, but with limited budget and on-the-ground capabilities; ‘Medicine Across Borders’, a non-governmental organization advocating for, and often leading emergency health interventions; and ‘the Foundation’, a well-resourced private foundation. Each actor team was provided with a description of their interests, capabilities, and relationships with other actors. The actors shared a common goal — to contain an influenza outbreak — but not a complete ‘harmony of interests’, which meant that competition and coordination problems could occur despite a backdrop of international cooperation.

The game unfolded through a series of rounds: the first round was an international preparedness conference for a global health crisis; the second round experienced a localized influenza outbreak in Eastland; the third round experienced its spread globally; and in the fourth round the virus was contained. Each round began with ‘news injects’ projected onto a screen, presenting the state of the outbreak and broader context. In addition to headlines, tweets highlighted perspectives from social movements. During each round, the teams took turns making an argument comprising of an action (e.g., deploy the military), the intended result of that action (e.g., containment), and the reasons why it would succeed (e.g., military well-resourced). Other participants could add additional reasons for (e.g., may prevent spread across borders) or against (e.g., fear of military may compel those infected to hide) the argument. Taking these arguments into consideration, the ‘gamemaster’ then determined the likelihood of success and rolled the dice. In between rounds the gamemaster updated the news injects and state of the outbreak based on players’ actions. The game ended with a debriefing discussion and a follow-up survey.

### Experiencing prioritization & co-operation challenges

**Fig. 2:**
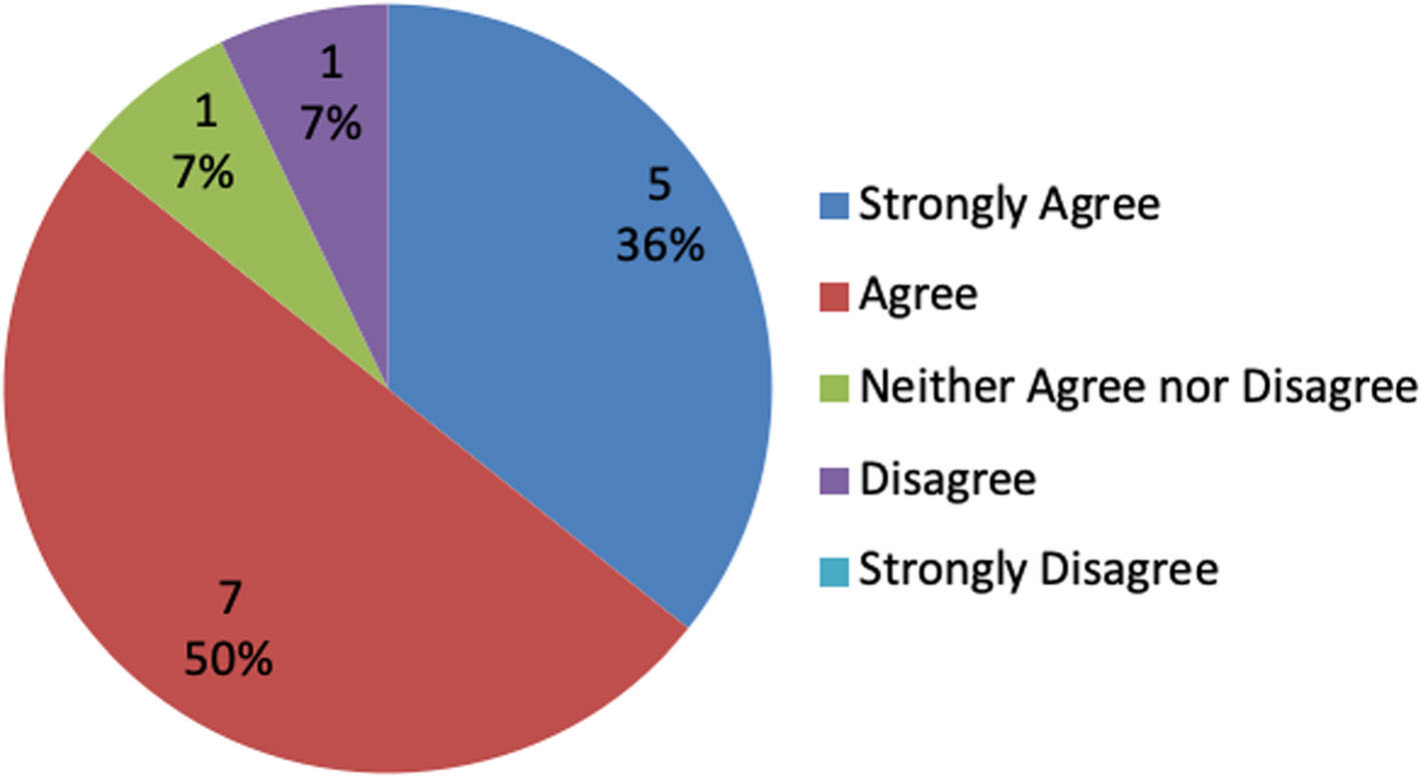
Matrix games like this one are useful tools for foreign policy planning and development

**Fig. 3:**
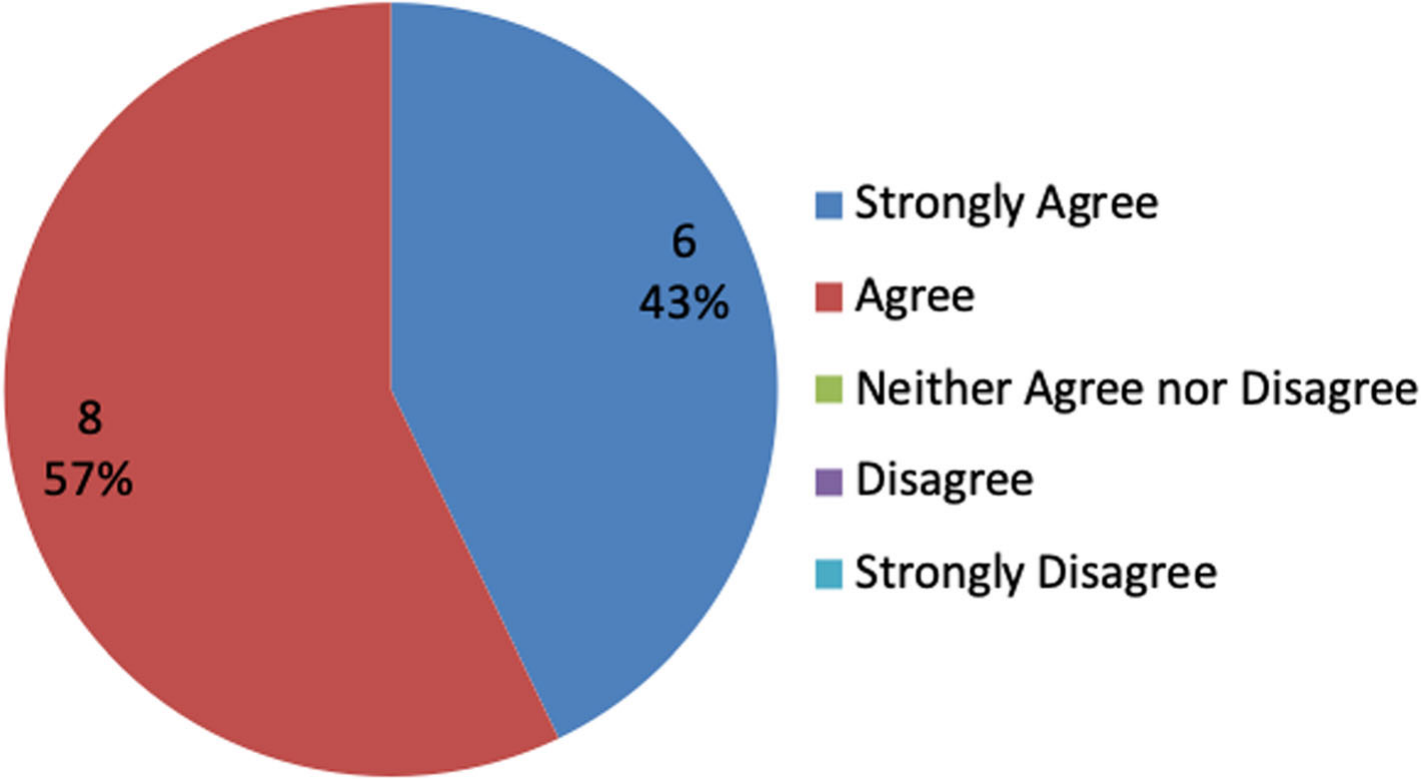
Matrix games like this one are useful tools for thinking about GAC’s response to an international crisis scenario

The survey asked if participants found the game useful for foreign policy planning and for thinking about an international response to a global health crisis. In both cases the majority of participants agreed with these statements (see Figs. 2 and 3). Open ended responses indicated that the matrix game format was reflective of decision-making contexts, noting it emphasized, ‘The importance of thinking on your feet.’ Another respondent wrote, ‘We can try to predict how certain policy positions will play out - but there are many factors - including the roll of the dice (unpredictability) that can influence. This is a very good reflection of today’s world.’ While players expressed frustration at having to prioritize certain actions, make decisions quickly and respond to unpredictable developments, it was recognized that these frustrations were reflective of actual policy contexts. Observers felt that the progress of events mirrored real interactions, including tugs-of-war over funding and tensions between regions.

Five (out of 12) responses to the survey question ‘What was your biggest take away from the game?’ mentioned lessons related to co-operation. One respondent wrote ‘The importance of demonstrating why the position I/my organisation is taking is in the interests – at least to some degree – of the others with whom I am engaging. The actors in the game too quickly fell into a competitive mode if they didn’t understand how interests were aligned.’ Another wrote, ‘We learned the importance of respecting the motives and goals of state and non-state actors during interactions; how easy it can be to “lose” potential partners and how difficult it can be to regain them.’ Participants experienced and learned from the tensions that emerge between the pursuit of individual actor interests and the need to co-operate in response to a health crisis.

Respondents also commented on the inclusion of non-state actors in the game: ‘I think it was a really good idea to have a company foundation represented in this game. More and more international cooperation is looking to partner with the private sector. But the private sector has its own agenda, and one that we traditionally would not have considered in a global pandemic scenario. Including this non-state actor provided an opportunity to think through how they would act in a crisis and how we, as the government, would likely react was really valuable.’ The participants who played the Foundation role noted that it was challenging to separate its commercial interests from its charitable activities, which they felt was an authentic experience.

### Outbreak preparedness

**Fig. 4:**
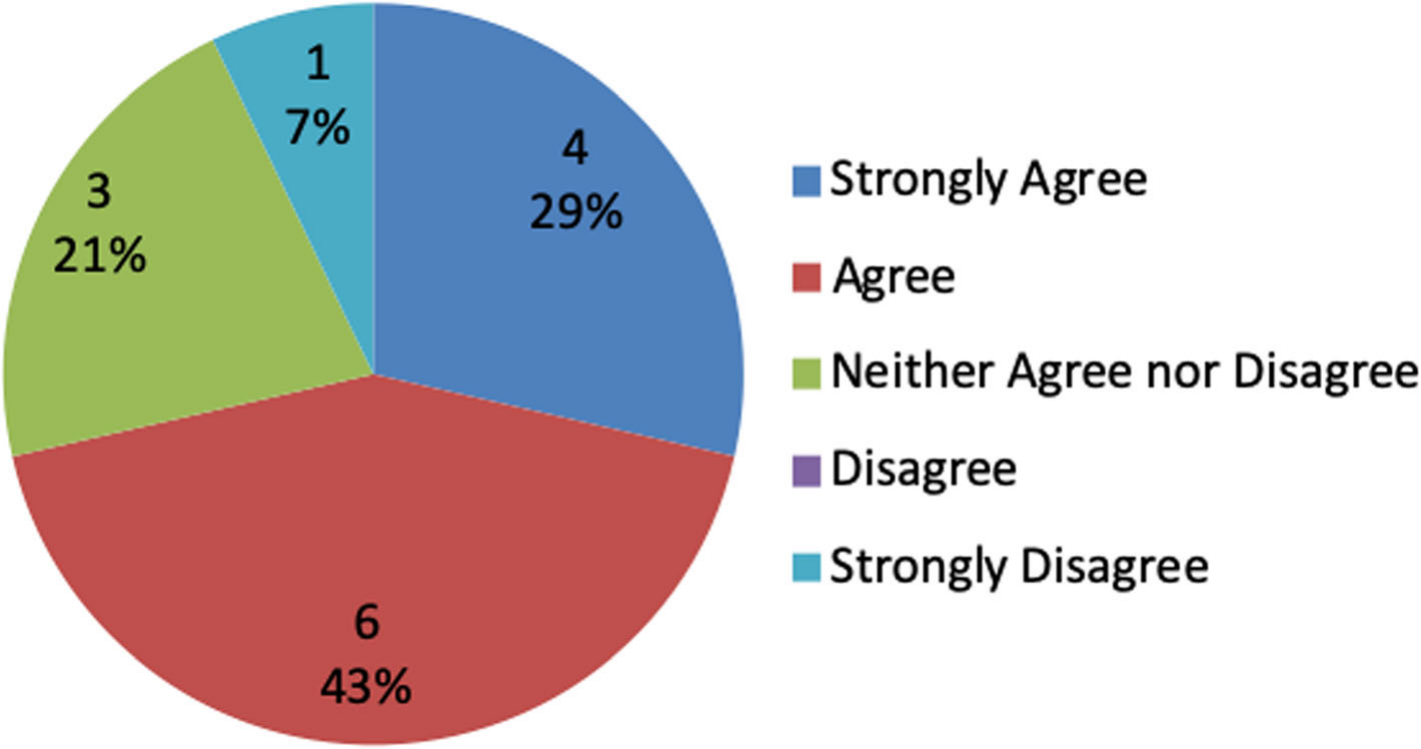
After playing this game, I now know more about the issues involved in ‘preparedness’ for a global health and security crisis, such as pandemics

Recognizing that lack of outbreak preparedness is a prominent global health challenge [iii], one of the objectives of the exercise was to encourage critical thinking about preparedness for a global pandemic scenario. Overall, the majority of players felt they knew more about preparedness after playing the game, though the challenges of preparing for an unknown outbreak also resulted in almost a third disagreeing (see Fig. 4).

During the first round of the game, teams were told they were attending an international conference on preparedness, but had no knowledge of the crisis that would eventually unfold. Through their actions they determined the priorities of the conference - mobilizing resources for vaccine development and an emergency response fund. There was little discussion of preventing or preparing to address the social determinants of outbreaks, other than earing-marking some funding for GBA+ initiatives, though what these would be was not clarified. During post-game reflections participants noted that the early focus on vaccine development, which continued throughout the game, led to neglect of other aspects to preparedness. Participants were particularly frustrated that by the time they succeeded in developing a vaccine (during the third round), the outbreak had spread exponentially and lack of communication had led to vaccine hesitancy among vulnerable groups. This led to further discussions on why such aspects were deprioritized over technical solutions.

Many players also noted that it was challenging to come to agreements with other actors during the preparedness stage of the game, with seven out of the 10 complete answers to the question on what was learned about preparedness again focusing on cooperation. For example, ‘GH [global health] preparedness requires input from multiple stakeholders domestically and internationally. Galvanizing this prior to a crisis is extremely challenging.’ And another respondent noted, ‘Relations are important. It is important to understand everybody’s interest and vision.’ Post-game discussions included recognition that while cooperation was challenging during the preparedness round, as the game progressed and the crisis situation heightened, actors became more willing to cooperate. Respondents recognized that if greater cooperation had been achieved during the preparedness conference the response to the epidemic would have been more effective.

### (not) applying gender-based analysis

The game explicitly aimed to incorporate gender-based analysis in recognition of Canada’s Feminist International Assistance Policy, which aims, among other goals, to ‘focus its efforts on programs and projects that put gender at the heart of their efforts to improve health care. .. [including] initiatives that help fight infectious diseases through equity-based approaches’ [9]. To support feminist policy implementation, Status of Women Canada developed the Gender-based Analysis Plus (GBA+) framework. GBA+ ‘is an analytical tool used to assess how diverse groups of women, men and gender-diverse people may experience policies, programs and initiatives’ [ix]. It was expected that as most players were employees of the Government of Canada, which has dedicated substantial resources and training programs to promoting GBA+, they would have some previous knowledge of and exposure to GBA+.

GBA+ was incorporated into game development through the inclusion of incentives and prompts for players to consider how different genders, sexualities, races and other social groups experience disease outbreaks and responses, and to recognize the social-determinants of health more broadly. Each actor package included guidance on GBA+ approaches, and each round of the game included news injects and tweets highlighting how the outbreak and response was affecting different genders and social groups. During debates over actions, a subject matter expert presented GBA+ arguments for or against actions.

However, players only sporadically incorporated GBA+ and rarely took actions to address the social determinants of health. Of the 24 actions proposed by actors, only seven (29%) included a GBA+ element. Three included support for maternal and child health, with two simply noting a GBA+ lens would be applied to other actions, and one noting that social and cultural characteristics would be taken into consideration when developing awareness materials. Survey respondents commented on the lack of integration of GBA+, with one writing, ‘I felt like this exercise didn’t touch upon GBA+ issues as much as I would have hoped - although there were some mentions of it, we didn’t have a chance to really explore it.’

Despite lack of application, the majority of players agreed that the game promoted critical reflection on applying GBA+ (see Fig. 4). One survey respondent noted, ‘My biggest takeaway was the difficulty in making decisions through a GBA+ lens and that political considerations are always prevalent, even during a humanitarian crisis.’ The lack of opportunity to apply GBA+ was attributed by respondents to the game structure, which allowed one action per turn, simulating the real experience of priority setting. It was noted that while players included GBA+ in their internal team discussions, it was rarely prioritized when it came to proposing actions. A survey respondent noted, ‘As usual, when we want to act quickly on an issue, we often think the GBA+ consideration is less important, which results in bad planning.’ Others noted that they did not emphasize GBA+ because they felt it made cooperation with other actors more difficult.

**Fig. 5:**
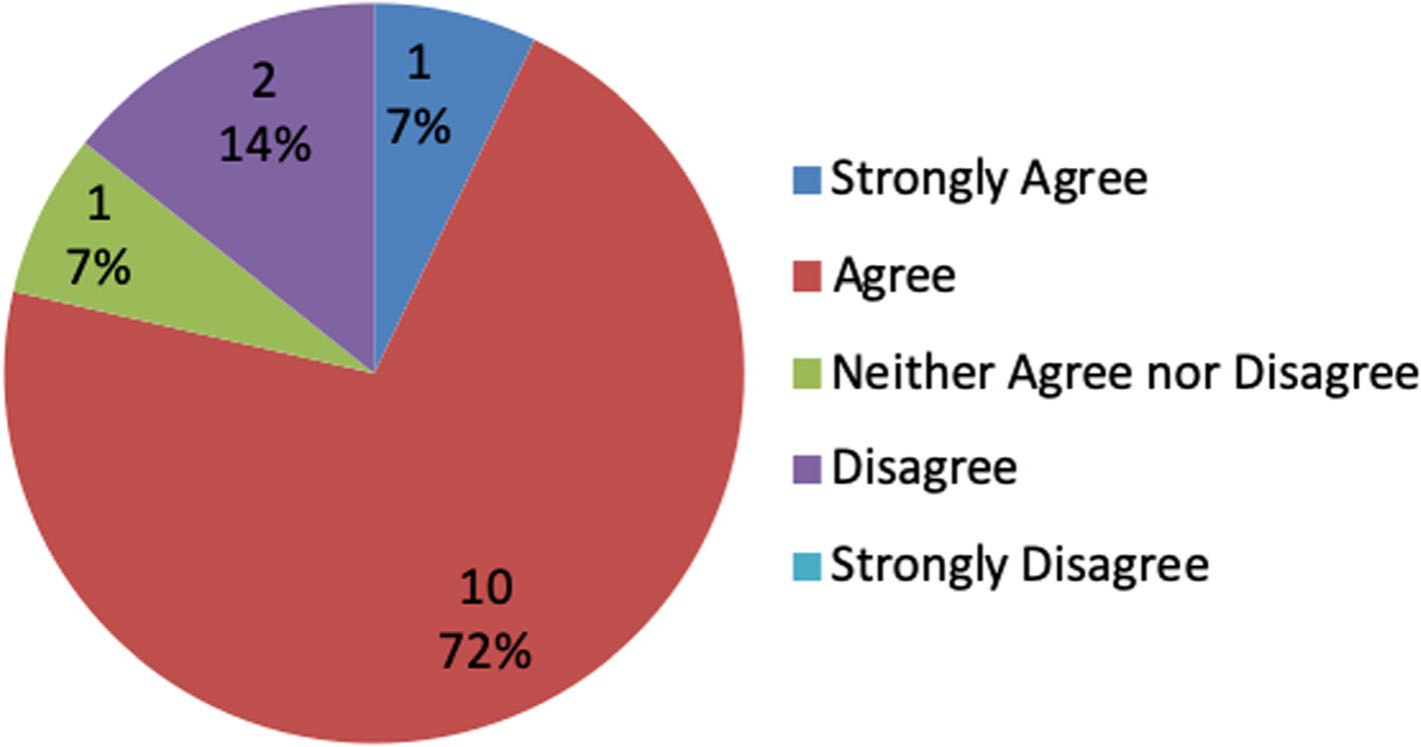
Matrix games like this one are useful tools for thinking about GAC’s implementation of the GBA+ framework in foreign policy

Such responses suggest that many of the challenges documented regarding limited application of gender-analysis during crisis response, such as it being de-prioritized compared to technical solutions [ii], were experienced and reflected on by the players. The final report on the exercise by GAC noted, ‘With the knowledge that outcomes for women in situations of crisis are significantly worse when gender needs are not incorporated into disaster responses, watching it play out in a fictional scenario (when participants are supposed to be doing the opposite) is a red flag that more understanding is needed on how this phenomenon is replicated and that specific guidelines and oversight are needed to adequately address the issue’ (Fig. 5) [10].

## Conclusion

Ongoing and emerging infectious disease outbreaks around the world indicate the urgent need for innovative and critical thinking in response to global health crises. Our experience suggests that ‘serious games’ can promote innovation and learning around health policy and global crisis response for experts, those new to the field, and those working in related sectors, such as security. Survey responses and participant reflections indicate the exercise met, to varying degrees, the objectives set out by GAC/DND to assess the utility of (matrix) games as a foreign policy learning tool; encourage critical thinking about preparedness; and promote the application of GBA+. In particular, by promoting internalized learning around how neglecting the social determinants of health, such as gender inequities, limit effectiveness, serious games have the potential to promote more transformative global health responses.

Two key learnings emerge from this game related to infectious disease preparedness and response. First, the co-operative element of the game created a particular challenge, with post-game reflections focusing on navigating tensions between individual, often competing, interests and the need to collaborate, often with the private sector, in order to mobilize an effective global health response. Participants identified a shift in incentives to cooperate as the game progressed from preparedness to crisis response, and how this impacted the success of their actions. This outcome suggests the need for global health actors to explore ways to translate these during-crisis incentives for cooperation into pre-crisis preparedness.

Secondly, it is apparent that (even for those with past experience and training) gender-based analysis takes practice. While the inclusion of GBA+ in game design did not promote the degree of application hoped for, the game offered an opportunity to reflect on the ways in which the social determinants of health are put aside during a crisis scenario, why this happens, and to critique these processes. Participants experienced the barriers to prioritizing social over technical responses, implementing GBA+ and the consequences of failing to do so. Global health organizations and governments need to not only provide resources, but also engage staff in opportunities to apply gender-based analysis and internalize learning.

This is but one example of a matrix game applied to a global outbreak. Serious games can take many forms, depending on the particular questions and objectives that inform game design. For instance, the scenario in this game design made an outbreak inevitable; it therefore did not allow much opportunity for consideration of prevention, beyond those measures related to preparedness. Matrix games could be employed to tackle the question of prevention, for instance, through a scenario with multiple rounds of actions on prevention and where in the final round the adjudication would determine the likelihood of an outbreak. There are countless additional ways games could be adapted to varying global health challenges.

The overwhelmingly positive response to our game from participants — ranging from graduate students to high-ranking public servants — suggests that serious games are suitable for a wide audience. While some games may require expensive technology and specific expertise, most can be implemented with few resources and by those with basic knowledge of the game format and the topic being explored. Due to their flexibility, adaptability and accessibility, serious games offer a potentially powerful tool to promote innovation and learning amongst global health practitioners and policymakers. However, there is only a very limited literature on the use of serious games in general and none, to our knowledge, on their application to global health. This commentary has aimed to make a first step to rectifying this gap by documenting our experience in hopes of informing further development of games as a learning and research tool in global health.

## Data Availability

The gaming materials used and data analyzed during the current study are available from the corresponding author on reasonable request.

## Abbreviations

DND: Department of National Defense
DRC: Democratic Republic of Congo
GAC: Global Affairs Canada
GBA +: Gender-Based Analysis plus

## Footnotes

1 Pangea is the name given to the supercontinent that existed around 200–300 million years ago
